# Inversion of chlorophyll content under the stress of leaf mite for jujube based on model PSO-ELM method

**DOI:** 10.3389/fpls.2022.1009630

**Published:** 2022-09-30

**Authors:** Jianqiang Lu, Hongbin Qiu, Qing Zhang, Yubin Lan, Panpan Wang, Yue Wu, Jiawei Mo, Wadi Chen, HongYu Niu, Zhiyun Wu

**Affiliations:** ^1^College of Electronic Engineering, College of Artificial Intelligence, South China Agricultural University, Guangzhou, China; ^2^National Center for International Collaboration Research on Precision Agricultural Aviation Pesticide Spraying Technology (NPAAC), Guangzhou, China; ^3^Guangdong Laboratory for Lingnan Modern Agriculture, Guangzhou, China; ^4^Key Laboratory of Digital Earth Science, Aerospace Information Research Institute, Chinese Academy of Sciences, Beijing, China; ^5^The 14th Division of Xinjiang Production and Construction Corps, Institute of Agricultural Sciences, Kunyu, China

**Keywords:** SPAD, PSO-ELM, SPA, hyperspectral, jujube, damage severity, leaf mite

## Abstract

During the growth season, jujube trees are susceptible to infestation by the leaf mite, which reduces the fruit quality and productivity. Traditional monitoring techniques for mites are time-consuming, difficult, subjective, and result in a time lag. In this study, the method based on a particle swarm optimization (PSO) algorithm extreme learning machine for estimation of leaf chlorophyll content (SPAD) under leaf mite infestation in jujube was proposed. Initially, image data and SPAD values for jujube orchards under four severities of leaf mite infestation were collected for analysis. Six vegetation indices and SPAD value were chosen for correlation analysis to establish the estimation model for SPAD and the vegetation indices. To address the influence of colinearity between spectral bands, the feature band with the highest correlation coefficient was retrieved first using the successive projection algorithm. In the modeling process, the PSO correlation coefficient was initialized with the convergent optimal approximation of the fitness function value; the root mean square error (RMSE) of the predicted and measured values was derived as an indicator of PSO goodness-of-fit to solve the problems of ELM model weights, threshold randomness, and uncertainty of network parameters; and finally, an iterative update method was used to determine the particle fitness value to optimize the minimum error or iteration number. The results reflected that significant differences were observed in the spectral reflectance of the jujube canopy corresponding with the severity of leaf mite infestation, and the infestation severity was negatively correlated with the SPAD value of jujube leaves. The selected vegetation indices NDVI, RVI, PhRI, and MCARI were positively correlated with SPAD, whereas TCARI and GI were negatively correlated with SPAD. The accuracy of the optimized PSO-ELM model (*R*^2^ = 0.856, RMSE = 0.796) was superior to that of the ELM model alone (*R*^2^ = 0.748, RMSE = 1.689). The PSO-ELM model for remote sensing estimation of relative leaf chlorophyll content of jujube shows high fault tolerance and improved data-processing efficiency. The results provide a reference for the utility of UAV remote sensing for monitoring leaf mite infestation of jujube.

## Introduction

The jujube tree (*Zizyphus jujuba*), which plays a significant role in the ecological and economic development of the Xinjiang oasis, is a key component of agricultural growth in southern Xinjiang. One of the primary pests that endanger the health of jujube is the leaf mite (*Tetranychus truncatus Ehara*), and when it infests the jujube during the growth season, it can lower the quality of the jujube by more than 35%. Therefore, efficient pest control and early detection are crucial for jujube orchard management.

Remote sensing monitoring using low-altitude unmanned aerial vehicles (UAVs), such as UAVs equipped with multispectral and hyperspectral cameras, addresses the above issues. In addition to low-altitude UAVs, measurements using satellites are also available for remote sensing to monitor the growth of crop plants. In recent years, agricultural pest and disease monitoring has increasingly utilized remote sensing monitoring technology (Adao et al., 2017; Bai et al., 2020; Jiang et al., 2021). With its rapid, real-time, large-area, and non-destructive qualities, the technology has demonstrated benefits that cannot be matched by standard pest and disease monitoring approaches. Large-scale monitoring of crops, including crop area, pest and early warning, and growth conditions, may be accomplished by satellite remote sensing (Bai et al., 2019). However, throughout the imaging process, satellite remote sensing optical images are frequently influenced by inclement weather such as clouds, rain, and fog. Compared with satellite remote sensing, UAV remote sensing platforms have the characteristics of low operating cost, high flexibility, and fast data acquisition in real time, which is a unique advantage in the field of crop pest and disease detection. As an essential component of low-altitude remote sensing (Zhang et al., 2021), UAV remote sensing platforms have unique advantages for crop pest and disease monitoring, which considerably expands the scope of remote sensing use in crop monitoring (Dehkordi et al., 2020; Xu et al., 2022). Satellite remote sensing is primarily used for monitoring broad areas, but it cannot provide images with sufficient spatial resolution and the images are susceptible to weather conditions (Bendig et al., 2015; You et al., 2022). In addition, the progressive improvement of UAV technology has made feasible its combination with hyperspectral and multispectral technology for agricultural disease monitoring, providing a reference for accurate crop disease monitoring and to guide remedial management (Adao et al., 2017; Li et al., 2021). For instance, UAV hyperspectral remote sensing can monitor a broad area with high precision, efficiency, and continuity, and accomplish the fusion of UAV multisource remote sensing imagery and target extraction. In previous studies (Liu et al., 2020), UAV hyperspectral remote sensing has been utilized to perform pertinent research on a variety of agricultural diseases, such as citrus Huanglongbing (Garcia-Ruiz et al., 2013; Deng X.L. et al., 2020), wheat yellow rust (Dehkordi et al., 2020; Guo et al., 2021), and pine wilt disease (Deng X. et al., 2020; Qin et al., 2021; Xia et al., 2021), etc.

UAV hyperspectral remote sensing facilitates information extraction in image and spectral dimensions, and is frequently employed for monitoring agricultural growth conditions, and pest and disease stress in the field. Photosynthesis is an essential reference for evaluation of plant development (Hunt et al., 2013, Sun Q. et al., 2021), and chlorophyll content is an indication of plant photosynthetic capacity; hence, chlorophyll content can effectively reflect the growth status of a crop (Ji et al., 2021; Kaivosoja et al., 2021; Lei et al., 2021). The variation of the chlorophyll content of crops is important for monitoring the growth of crops. On the one hand, chlorophyll content absorption reflects the strength of photosynthesis, the growth stage and health status of crops; on the other hand, pests and diseases also directly affect the chlorophyll content of plants. Therefore, monitoring chlorophyll content effectively reflects the growth condition of crops. Variations in grayscale values on hyperspectral scanning recordings are caused on a broad scale when the crop is damaged by pests or disease, resulting in considerable variances in spatial, spectral, and temporal phases (Liu et al., 2017; Ahmad et al., 2018). The introduction of fused hyperspectral data and chlorophyll feature content approaches by analyzing local spectral differences of crops may also enhance remote sensing research on the monitoring of pests and diseases (Vanegas et al., 2018). It may be used for monitoring vegetation production, controlling crop resources, and monitoring pests and diseases by calculating the chlorophyll content of the crop canopy. Consequently (Wang et al., 2015), monitoring of crop chlorophyll content indicators might assist in reflecting the severity or incidence of agricultural pests and diseases to a certain extent.

A key biochemical indicator of crop development is chlorophyll content, and when jujube trees are infected with leaf mites, the amount of chlorophyll varies according to the degree of the disease. Hyperspectral has rich spectral information, which provides the possibility for the construction of chlorophyll inversion models. The severity of leaf mite infestation was correlated with chlorophyll content, which can be indirectly reflected by measuring the chlorophyll content of jujube. The majority of current research on crop chlorophyll inversion with hyperspectral data is based on statistical regression models, which may be broadly classified into two types: vegetation index models and direct spectrum models. In the vegetation index models, the hyperspectral data are first utilized to generate several vegetation indices (Sun Q. et al., 2021), which are then used to develop numerous linear or nonlinear regression methods to produce an inversion model between these indices and chlorophyll content in the vegetation index models (Guo et al., 2021; Ji et al., 2021; Sun J. et al., 2021). It is easy to build the inversion model using vegetation indices, but a single vegetation index cannot adequately characterize the entire hyperspectral information. The direct spectrum models rely on the modeling of the entire hyperspectral bands, which is usually a high-dimension vector. Using the whole hyperspectral band directly may result in excessive model complexity or model overfitting. Dimension reduction approaches like as principal component analysis (PCA) or partial least squares (PLS) may assist in addressing this issue in part.

Most of the current research on the relationship between hyperspectral data and chlorophyll content focuses on the changes in chlorophyll content of crops under different nutrient stresses and different growth periods, while the hyperspectral inversion research on chlorophyll content of crops infected by diseases and insect pests is relatively less. The main performance is that the research pays more attention to the spectral characteristics of crop diseases and less attention to the physiological and biochemical changes in plants caused by diseases and insect pests. In addition, the research on crop diseases and insect pests using remote sensing technology is mostly aimed at grain crops such as wheat and rice, as well as economic crops such as cotton, soybean, and rapeseed, which pay less attention to pests and diseases of jujube plants.

Therefore, the aim of this study was to estimate SPAD values for leaf mite infestation of jujube based on UAV hyperspectral images. The estimation performance of the model based on VIs and selected feature bands was also analyzed. The relationship between the degree of leaf mite infestation and canopy leaf SPAD values was investigated based on the best estimates of SPAD values obtained. More specifically, the following points were noted in our study:

1. Based on the experimental data, the correlation between the hyperspectral characteristic parameters of the jujube canopy and chlorophyll content was analyzed.
2. Establishment of jujube SPAD estimation model under stress of leaf mite based on VI alone by using a linear regression model.
3. To improve the accuracy of the inversion of the chlorophyll content of jujube infested with leaf mites. A proposed method employs a successive projection algorithm (SPA) to extract the characteristic bands from the high-dimensional hyperspectral vector, reducing model complexity and avoiding model overfitting. With the extracted characteristic bands as input, by building a PSO-ELM inversion model for the chlorophyll content of jujube.

## Materials and methods

### Study areas

The 224th regiment, the study area selected for this experiment, is located north of National Highway 315 at the crossroads of Pishan County and Moyu County in Hotan Region, on the southern edge of the Great Taklamakan Desert in Xinjiang, China (Li et al., 2021). The total land area is 234,751 km^2^ and the terrain slopes from the southwest to the northeast. Jujube predominates in the study area, which comprises a planting area of 74,057 ha, a sizable landmass, an abundance of light and heat resources, drought, low rainfall, high evaporation, low relative humidity, and significant diurnal temperature differences—all of which are unique natural conditions that have aided the explosive growth of the jujube industry in Xinjiang. The 14th division’s 224th regiment began planting jujube in 2003, according to investigations by the Xinjiang Production and Construction Corps. jujube orchards have expanded by more than 90 km^2^ since approximately 2019, and constitute 72% of all arable land and 83% of all orchard land (Liu et al., 2015).

**Figure 1:**
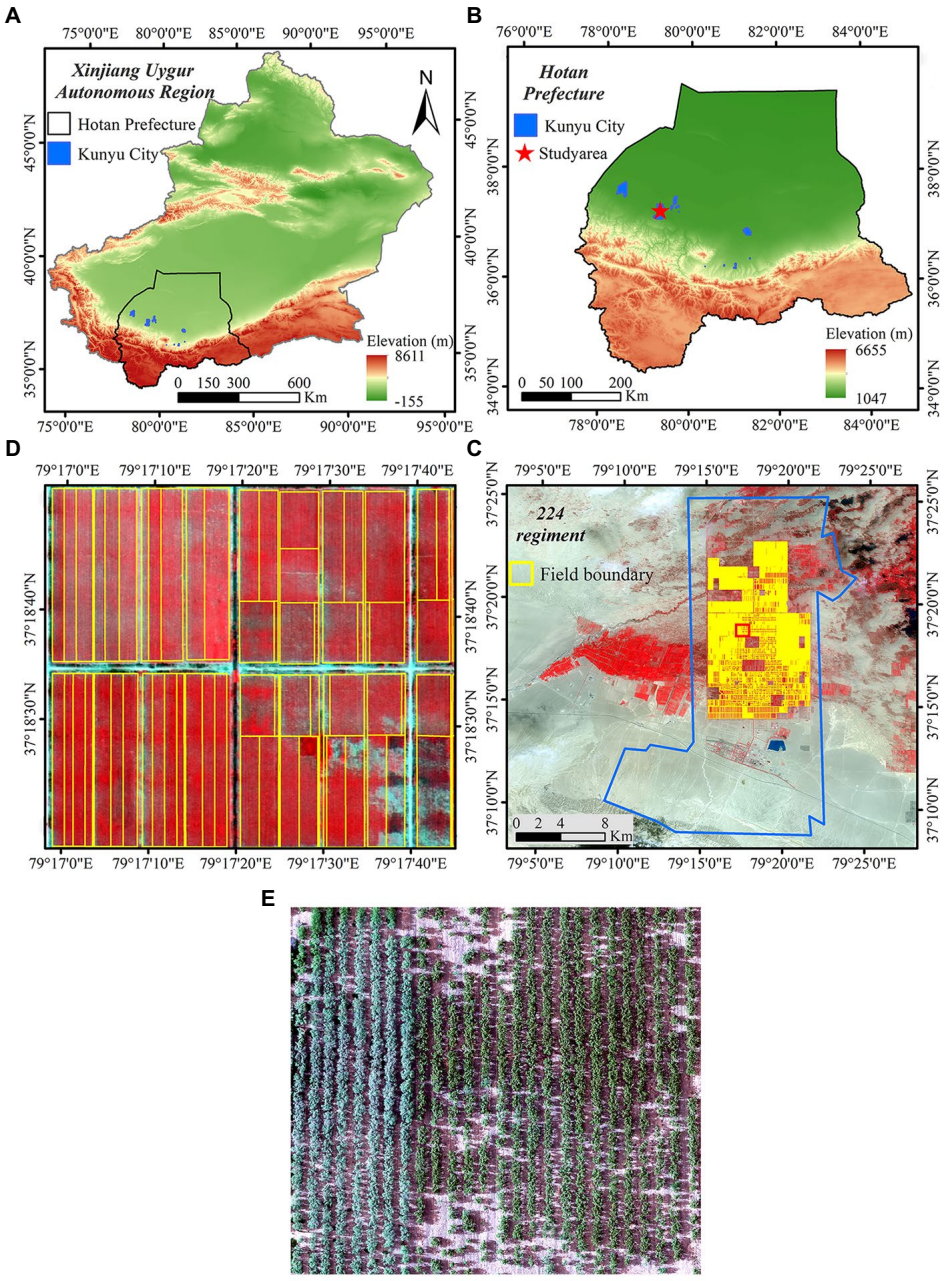
Study area. **(A)** Xinjiang Uygur Autonomous Region; **(B)** Hotan area; **(C)** 224th regiment; **(D)** and **(E)** Image of the study area.

At the three designated study areas, a total of 90 sample survey sites were selected, where communities of healthy jujube plants and plants infested with leaf mites were clearly separated. Taking into consideration the features of pest infestation and the distinguishability of remote sensing images, the infestation severity was divided into four classes: healthy, mild damage, moderate damage, and severe damage. Based on an investigation of the effects of environmental changes on leaf mite infestation of jujube trees in Xinjiang, it was determined that the peak incidence of leaf mites occurs annually from June to August (Zhang et al., 2013; Li H. et al., 2020). By clustering, leaf mites mostly suck sap on the underside of leaves, causing grayish white or yellowish fine patches on the leaves, decreasing the leaf chlorophyll content, and impairing the development and growth of jujube plants. In light of this, the present experiment chose the aforementioned period to conduct the research and employed an UAV-mounted hyperspectral sensor and ground acquisition for data collection in the field trial. The study area shown in Figure 1.

### Data acquisition

#### UAV hyperspectral remote sensing image acquisition and data processing

The experiment employed a M600Pro UAV (Shenzhen DJI, Shenzhen, China) equipped with a hyperspectral camera (Rikola, Oulu, Finland) and the SPAD-502Plus (Konica Minolta, Osaka, Japan). Supplementary Figure 1 depicts the experimental instruments and the scene diagrams. The acquisition period ranged from 11:00 to 15:00 (the sun altitude angle was >45°) under bright, clear, or partially overcast conditions. In anticipation of flight photography, radiation correction was conducted on the hyperspectral camera. Four 50 cm × 50 cm diffuse reflectance gray plates (reflections of 3%, 22%, 48%, and 64%, respectively.) were placed on a level surface in the test location, and the surface of the calibration plate was devoid of interfering objects and shadows. In accordance with the features of the hyperspectral imagers provided by Rikola, system correction and post-processing correction were conducted on the hyperspectral images after image acquisition was completed.

##### Correcting the system

In the course of capturing hyperspectral images, the UAV platform creates inevitable systematic inaccuracies owing to the instrument’s inherent constraints and the measurement technique, which must be addressed. Radiation calibration, dark current correction, and lens vignetting correction are the primary components.

The feature information of the original jujube tree orchard hyperspectral image was expressed as the digital number (DN). However, because the systematic error DN cannot accurately reflect the spectral characteristics of the feature, the DN of the original image must be converted to the feature reflectance using the information for the calibration plate representing the specific reflectance obtained at the same time as the experiment, as shown in Equation (1).


(1) $$\rho_{t}=\frac{DN_{t}-DN_{1}}{DN_{2}-DN_{1}}\left(\rho_{2}-\rho_{1}\right)+\rho_{1}$$

where$\rho_{t}$and $DN_{t}$ are the reflectance and DN of the original image target element, $\rho_{1}$ and $\rho_{2}$are the reflectance of different calibration plates, and $DN_{1}$ and $DN_{2}$ denote the DN value of different calibration plates, respectively.

##### Post-processing refinement

In this work, the UAV images were captured using frame-wide imaging. Owing to the imaging principle and environment, there are small changes in position and attitude between the bands, resulting in hyperspectral cube bands that do not totally overlap. The flight time of the UAV is ~20–30 min, and the radiation brightness gradient difference between different bands will be affected by the change of solar illumination conditions, resulting in inhomogeneous color and DN. The irradiance can be effectively corrected to the normal level using Equations (2), (3).


(2) $$L_{jc}{\left(\lambda \right)}_{\mathrm{at}\_\mathrm{sensor}}=L_{j}{\left(\lambda \right)}_{at_{\mathrm{sensor}}}\times C_{j}\left(\lambda \right)$$

(3) $$C_{j}\left(\lambda \right)=E_{j}\left(\lambda \right)/E_{ref}\left(\lambda \right)$$

where$L_{jc}{\left(\lambda \right)}_{\mathrm{at}\_\mathrm{sensor}}$is the irradiance consistency corrected image; $L_{jc}{\left(\lambda \right)}_{at_{\mathrm{sensor}}}$is the *j*th original image; $C_{j}\left(\lambda \right)$ is the *j*th image multiplicative correction factor; $E_{j}\left(\lambda \right)$ is the irradiance value recorded for the *j*th image; and $E_{ref}\left(\lambda \right)$ is the irradiance value of the reference image.

The UAV flew at a height of 60 m, at a speed of 5 m s^−1^, with overlap and side overlap of the images of 75%, a baseline distance of 25.9 m, a route spacing of 34.5 m. The Agisoft PhotoScan program was used to import photographs and the position and orientation system data, define the coordinate system, align the images, produce point clouds, grids, and textures, construct a digital elevation model, and produce orthophotos. The stitched orthophoto was geometrically corrected using GPS point data collected in the field to reduce the accuracy between the hyperspectral image features and the actual feature positions. The projection coordinate system was set to the Universal Transverse Mercator and the final correction error was controlled within 0.5 m. Within 0.5 m is the ultimate correcting error. Even after radiation correction, a variety of random disturbances remain in the picture reflectance, including impulse noise and Gaussian noise. Using Savitzky–Golay filtering, the spectral curve was considered to be polished, ensuring that the noise was efficiently smoothed with the same form and width as the signal.

### Measurement of SPAD at ground sampling points

The collection environment is shown in Supplementary Figure 2. A handheld chlorophyll absorbance meter, the SPAD-502Plus, was used to estimate the chlorophyll content of leaves swiftly and non-destructively. On the same day as the UAV flight, the SPAD properties of jujube trees were assessed. The field sampling points were arranged in the shape of a ‘S’, each of the three chosen blocks comprised 30 sampling points. Four classes of jujube trees were selected with the same spatial distribution. Thus, 90 sets of samples were gathered, consisting of a total of 1,200 samples. Following the sample allocation concept, 20 of the 90 groups of samples were utilized as test samples, while the measured SPAD values of the remaining 70 groups were randomly chosen as modeling samples. To minimize sampling error, canopy leaves of comparable size, color, and shape were chosen for the sampling procedure (Han et al., 2021). The measurements were performed at the leaf tip, center, and base, and the mean value was used to represent the leaf’s SPAD characteristic parameter.

### Classification of plant pest severity

**Figure 2:**
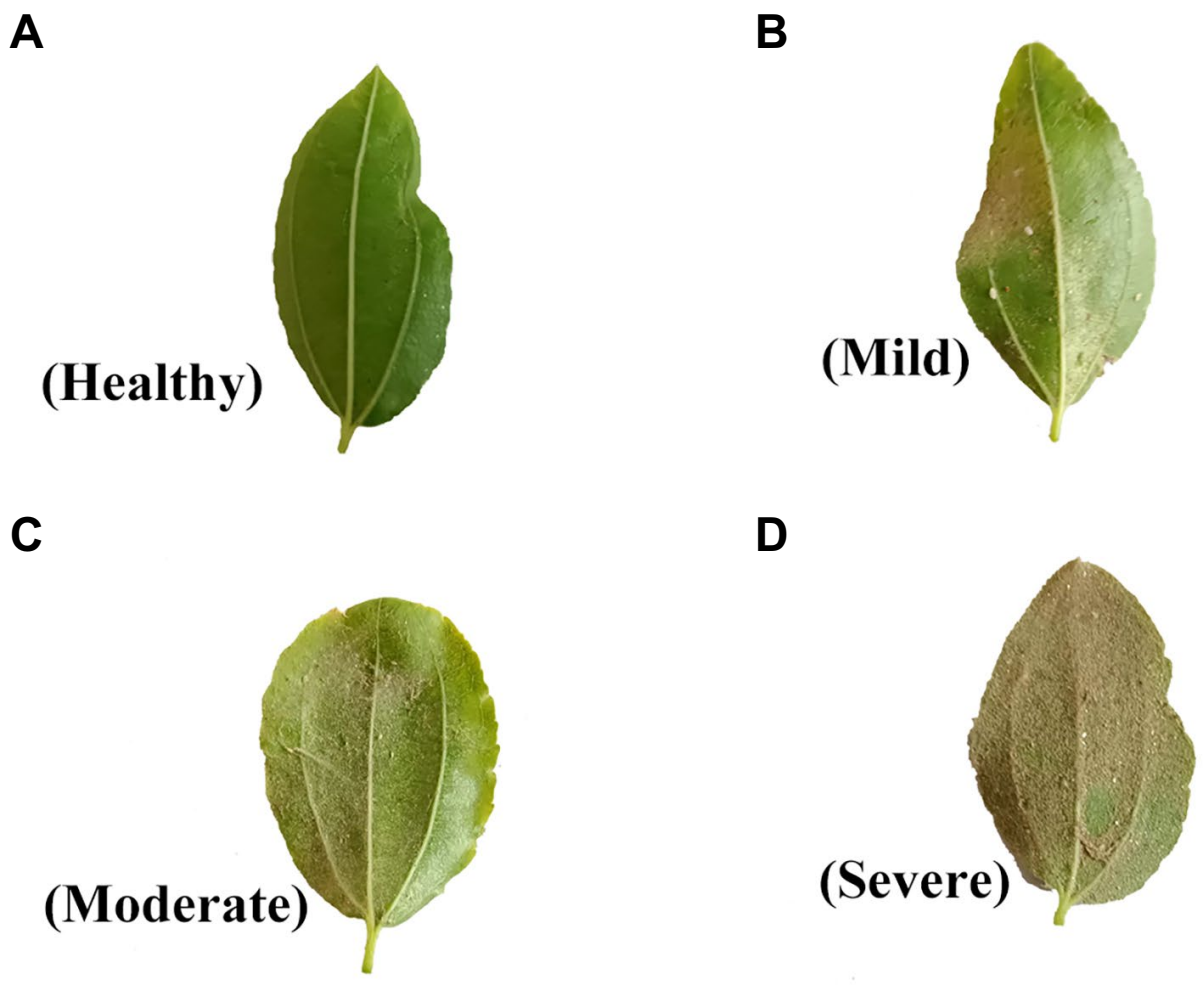
Different degrees of leaf mite infestation in jujube severity.

This study was carried out in experimental plots with leaf mite occurrence in the field, and field leaf mite surveys were conducted by hand to collect samples. At the time of sampling, the degree of leaf damage and the latitude and longitude information of the sampling site were recorded based on GPS positioning, the 90 sample points were sited evenly throughout the jujube tree planting area. According to the Code of Practice of Prevention and Control Techniques for Pests and Diseases of Jujube (National Standard of the People’s Republic China), the severity levels of jujube tree mite infestation was divided into four classes in Supplementary Table 1. Healthy leaves were assigned a value of I, mild damage a value of II, moderate damage a value of III, and severe damage a value of IV. The four categories leaves are shown in Figure 2.

### Vegetation index

A vegetation index may be subdivided into several vegetation index parameters based on various monitoring and computation methodologies (Torres-Sanchez et al., 2014; Liu et al., 2020; Ji et al., 2021). A vegetation index incorporates linear or nonlinear combinations of reflectance in distinct spectral bands to produce correlated spectral signals so as to simplify the spectral information and enhance vegetation-related features. For identification of agricultural pests and diseases, the visible red band, which is highly absorptive in green plants, and the near-infrared band, which is highly reflective and transmissive in green plants, are often selected. The spectral response of these two bands to the same biophysical phenomena provides a strong contrast that changes with the leaf canopy structure and coverage; hence, their ratio, difference, or linear combination may be utilized to augment or disclose the implicit vegetation information (Lei et al., 2021). In the present study, the normalized difference vegetation index (NDVI), ratio vegetation index (RVI), physiological reflex vegetation index (PhRI), modified chlorophyll absorption ratio index (MCARI), transformed chlorophyll absorption ratio index (TCARI), and green index (GI) were chosen. Information on the vegetation indices is presented in Table 1.

**Table 1:** Vegetation index information.

Name	Formula	Comprehensive embodiment	Application	Reference
NDVI	$\frac{NIR-R}{NIR+R}$	Integrated crop growth variability	Diseases detection	Mahlein et al. (2013)
RVI	$\frac{NIR}{R}$	Crops growth sensitivity	Chlorophyll estimation	Birth and McVey (1968)
PhRI	$\frac{\left(R_{550}-R_{531}\right)}{\left(R_{550}+R_{531}\right)}$	Crop growth pattern	Chlorophyll estimation	Daughtry et al. (2000)
MCARI	$\frac{\left(\left(R_{701}-R_{671}\right)-0.2\left(R_{701}-R_{549}\right)\right)}{\left(\frac{R_{701}}{R_{671}}\right)}$	Crops chlorophyll variations	LAI and chlorophyll estimation	Zhang et al. (2019)
TCARI	$3\left(\left(R_{700}-R_{675}\right)-\frac{0.2\left(R_{700}-R_{500}\right)}{\left(\frac{R_{700}}{R_{670}}\right)}\right)$	Crops growth sensitivity	Chlorophyll estimation	Haboudane et al. (2002)
GI	$\left(\frac{R_{554}}{R_{677}}\right)$	Crops green variability	Leaf rust detection	Ashourloo et al. (2014)

### Statistical analysis

Regarding the accuracy of the parameter estimates, the coefficient of determination (*R*^2^) and root mean square error (RMSE) were employed to assess the model accuracy. The *R*^2^ value represents the degree of fit, whereas RMSE measures the accuracy of data measurement. In general, it is believed that the closer the *R*^2^ value is to 1, the better it indicates a strong goodness of fit, and conversely, a low value indicates a poor goodness of fit. The smaller the RMSE, the better it indicates a small error, whereas a high value indicates the inaccuracy is large. The calculation of these statistics is shown in Equations (4), (5):


(4) $$R^{2}=\frac{\sum_{i=1}^{n}{\left(x_{i}-\hat{x}\right)}^{2}{\left(y_{i}-\hat{y}\right)}^{2}}{\sum_{i=1}^{n}{\left(x_{i}-\hat{x}\right)}^{2}\sum_{i=1}^{n}{\left(y_{i}-\hat{y}\right)}^{2}}$$

(5) $$\mathrm{RMSE}=\sqrt{\frac{\sum_{i=1}^{n}{\left(y_{i}-\hat{y}\right)}^{2}}{n}}$$

where n denotes the number of samples for estimation or validation of the model; $x_{i}$, $\hat{x}$,$y_{i}$, and $\hat{y}$ denote: measured value, measured mean value, estimated value, and estimated mean value, respectively.

## Results

### Characteristics of SPAD variation

From 90 sample points, a total of 1,200 ground SPAD values were obtained, Table 2 summarizes the statistical properties of the sampled data. The modeling sample and the validation sample differed except for the data samples. The variation range of SPAD values for the modeling set of samples was 20.80–66.90, the mean was 46.21, and the coefficient of variation was 21.01%. The variation range of SPAD values for the validation set of samples was 21.50–67.50, the mean was 45.97, and the CV was 21.06%. Considering the impact of leaf mites on the leaf chlorophyll content, the CV of the SPAD values was more than 10%, suggesting that the chlorophyll content was more variable. The discrepancies between the modeling and validation sets were negligible, there were no significant differences within the modeling and validation sets (*p* = 0.678), as determined by an independent samples *t*-test. Therefore, the sample sets were appropriate for modeling and validation.

**Table 2:** Statistical characteristics of chlorophyll content.

Sample set	No. of samples	Min.	Max.	Mean.	Std. deviation	C.V/%
Overall	1,200	20.80	67.50	46.17	9.66	20.93
Modeling Set	800	20.80	66.90	46.21	9.71	21.01
Validation Set	400	21.50	67.50	45.97	9.68	21.06

### Analysis of SPAD and spectral characteristics of jujube under infestation of leaf mite

**Figure 3:**
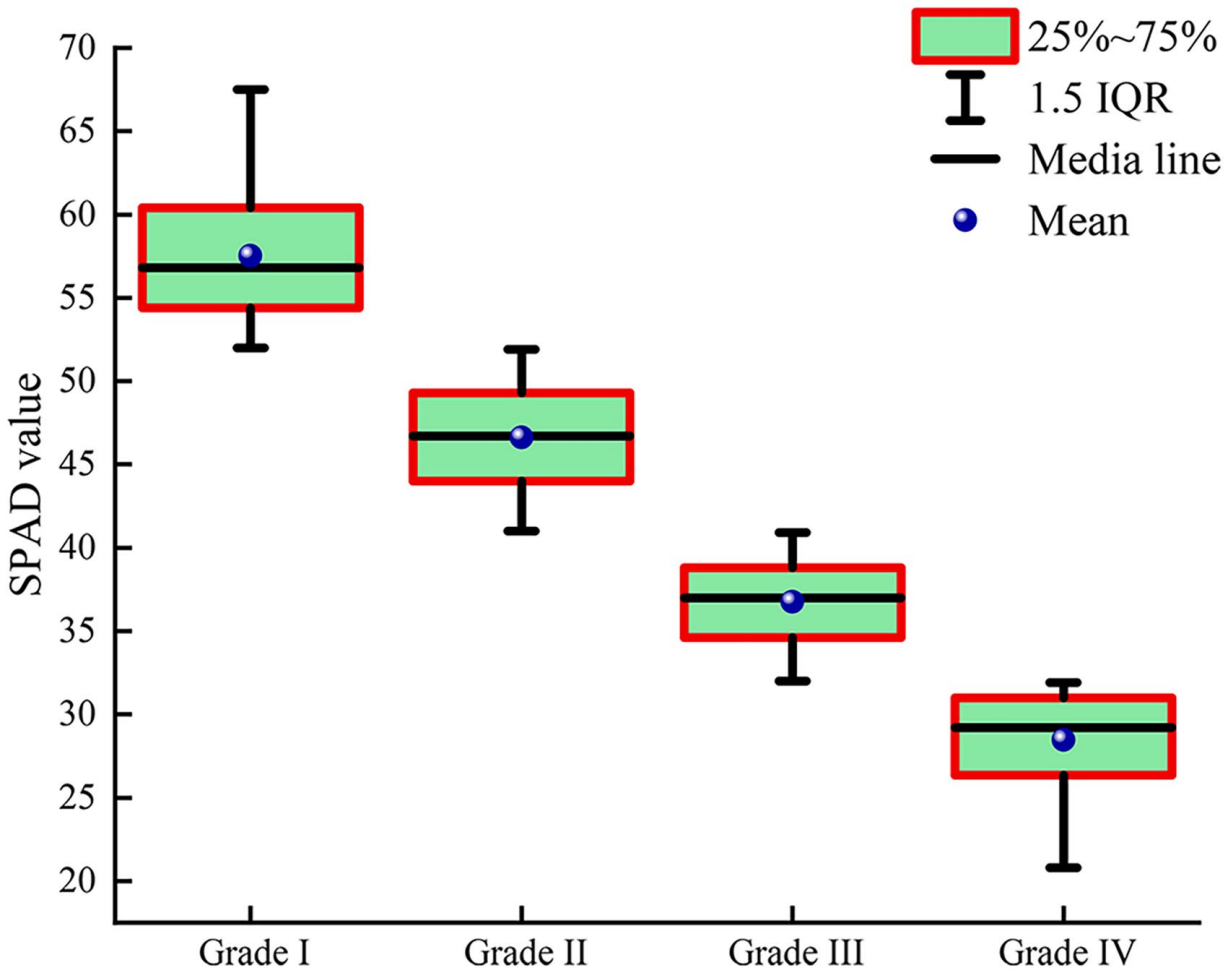
Variations in SPAD values of jujube leaves for different leaf mite infestation levels.

Chlorophyll content is an indicator of the biochemical parameters of the crop and reflects the growth of the crop (Qi et al., 2021). Pest infestation causes changes in the chlorophyll content of the crop. Thus, measuring chlorophyll content reveals the health and vigor of the crop. When jujube plants are infected with leaf mites, the mean SPAD value of their canopy leaves decreases gradually with an increase in the severity of leaf mite infestation (Figure 3). The results demonstrated that the SPAD value of jujube trees was negatively associated with the severity of leaf mite infestation.

**Figure 4:**
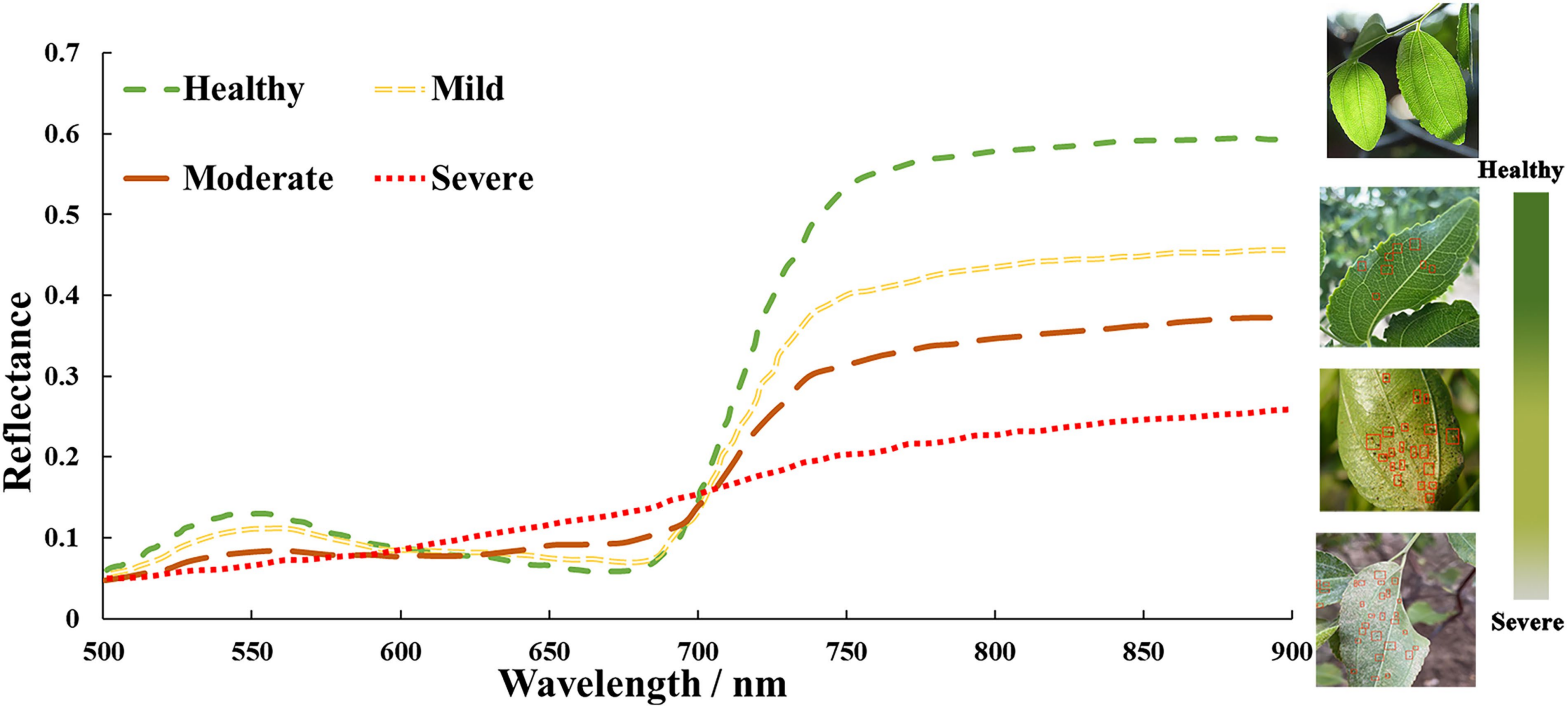
Spectral curves of jujube trees for different leaf mite damage indices.

With the more severe leaf mite infestation, the SPAD values of jujube chlorophyll content gradually decreased, thus causing changes in the spectral characteristics of the canopy of jujube, showing a trend of decreasing spectral reflectance step by step with the increase of leaf mite infestation. Figure 4 depicts the average spectral reflectance curves of jujube trees at the canopy scale under different severities of leaf mite infestation. The spectral band features of jujube plants differ notably with the severity of leaf mite infestation. Considering the phenomena of “green peaks” owing to decreased chlorophyll absorption, the spectral characteristic curves of healthy jujube trees exhibited modest reflectance peaks in the green band between 520 and 570 nm. Because of the intense absorption of chlorophyll for photosynthesis, a red wavelength absorption trough, termed a “red valley,” forms in the red wavelength range of 620–690 nm. As the chlorophyll concentration rises, so does the photosynthetic capability. The “green peak” and “red valley” in the green light spectrum progressively diminish between 680 and 750 nm. Given light scattering within the leaf, the reflectance in the near-infrared range exhibits conspicuous peaks of high reflectance, which constitute the spectrum’s largest peak and generate a highly reflective platform. The variation in spectral reflectance of leaf mite damage of jujube trees was increasingly evident with an increase in the severity of infestation, which led to a decline in chlorophyll content and severe damage to the cellular structure and tissues of the leaf.

### Correlation between SPAD value and vegetation indices of jujube trees

**Figure 5:**
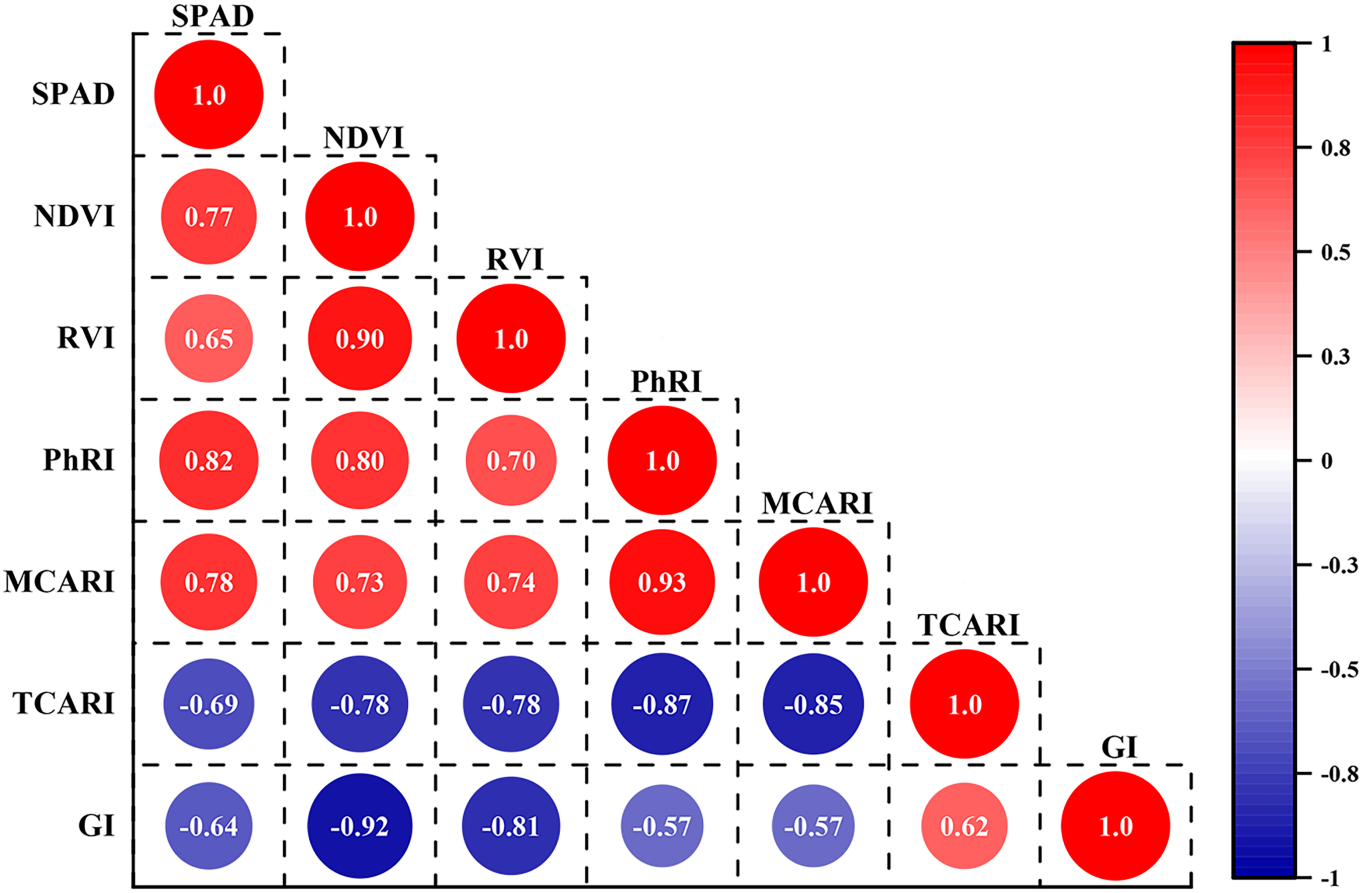
Correlation analysis between SPAD and vegetation index.

To facilitate an understanding of the relationship between vegetation indices and the chlorophyll content of jujube, a correlation coefficient matrix map is presented in Figure 5. Positive correlations are represented by numbers greater than zero, whereas negative correlations are represented by values less than zero (Yang et al., 2021). The absolute values of the correlation coefficients between SPAD and NDVI, RVI, PhRI, MCARI, TCARI, and GI ranged from 0.64 to 0.82. The NDVI, RVI, PhRI, and MCARI were positively correlated with SPAD, whereas TCARI and GI were negatively correlated with SPAD. As can be seen in Figure 5, the six selected vegetation indices were significantly correlated with SPAD, among which the correlation coefficient between leaf SPAD value and PhRI reached a maximum of 0.82, which was higher than the correlation coefficient between SPAD value and other vegetation indices. Further, by taking SPAD of jujube leaves as the dependent variable, and using NDVI, RVI, PhRI, MCARI, TCARI, and GI as independent variables, a remote sensing estimation model for the relative chlorophyll content of jujube canopy leaves was constructed. Table 3 shows the statistical regression modeling of vegetation indices to inversion chlorophyll content. The modeling determination coefficient of the SPAD-PhRI estimation model was 0.702, which was higher than the modeling accuracy of SPAD value and other vegetation indices.

**Table 3:** Correlation between SPAD values of canopy leaves and vegetation index of jujube trees.

VI	Model	*R* ^2^	RMSE
NDVI	*y* = 2.04*x* + 65.36	0.668	1.062
RVI	*y* = 18.14*x* + 101.76	0.585	0.951
PhRI	*y* = 15.61*x* + 94.95	0.702	0.886
MCARI	*y* = 2.0*x* + 65.3	0.657	0.869
TCARI	*y* = −0.53*x* + 80.20	0.632	0.896
GI	*y* = 2.74*x* + 72.55	0.608	0.787

### Correlation between SPAD value and spectral reflectance

**Figure 6:**
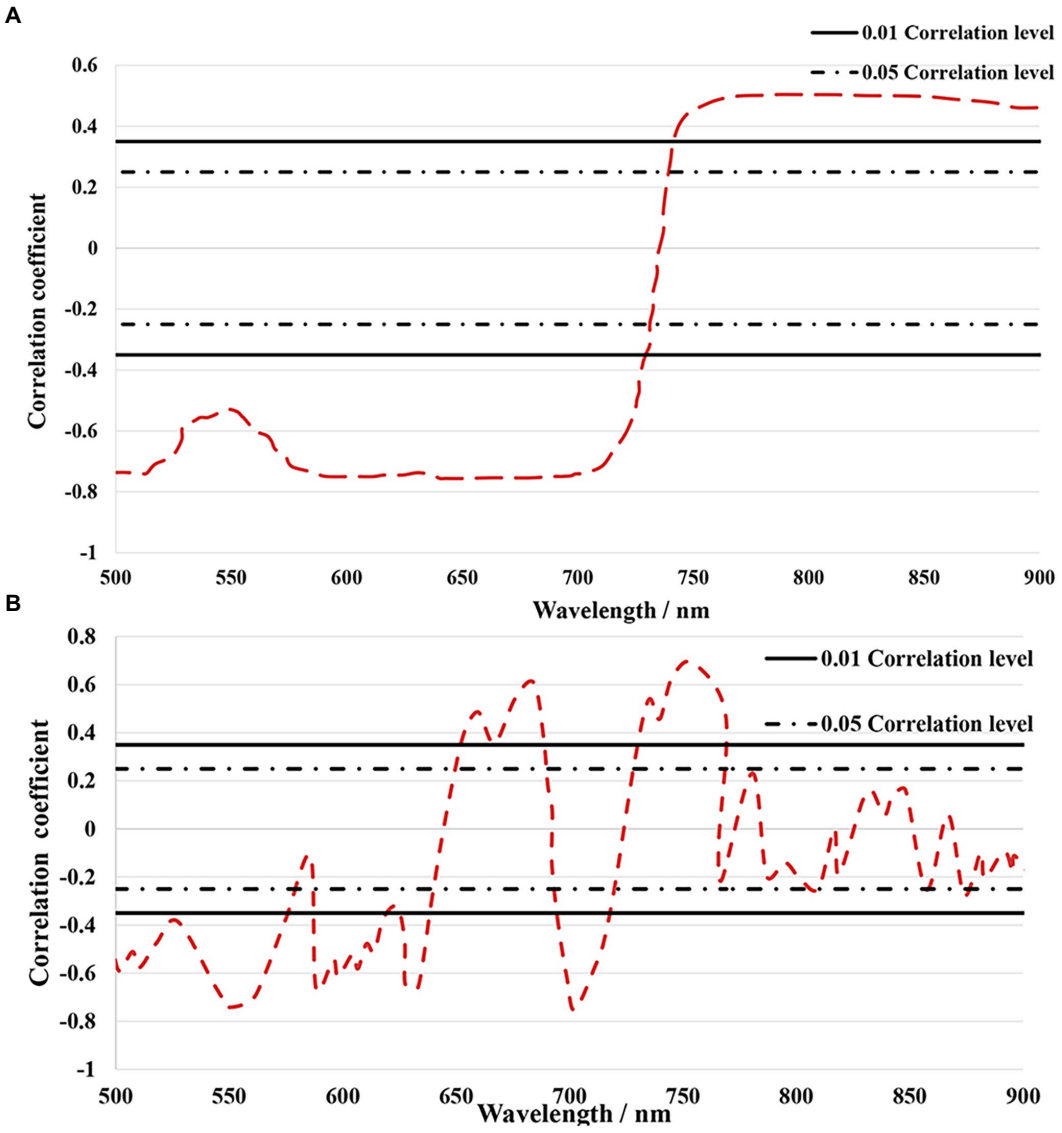
**(A)** Raw spectra with SPAD correlation analysis; **(B)** First-order derivative spectra with SPAD correlation analysis.

As the chlorophyll content of jujube trees infected by leaf mites will change, as illustrated in Figure 6, chosen chlorophylls significantly associated with leaf mite infection were correlated with the raw and first-order derivative spectra for the analysis. The correlation coefficients between the original spectra and the SPAD value were negative at 500–749 nm and positive above 750 nm (Figure 6A). The absolute value of the correlation between the original spectrum and the chlorophyll content is mostly between 0.5 and 0.65, and the curve changes are relatively flat. When the original spectrum is transformed by the first derivative, the correlation with the chlorophyll content of jujube leaves is significantly enhanced in some wavelength bands, among which it reaches a very significant positive correlation at 660, 685, 735, and 754 nm, and at 550, 588, 633, and 702 nm highly significant negative correlation. The maximum correlation coefficients between the first-order derivative spectra and the SPAD value were −0.75 and 0.70 at 702 and 754 nm (Figure 6B), respectively. It is evident that the chlorophyll of jujube leaves strongly affects the first-order differential spectrum under the leaf mite infestation. The curve of the correlation coefficient between the first-order derivative spectrum and chlorophyll content fluctuates obviously. Considering that the spectral derivative enhances the slight change in the slope of the spectral curve, the reason for this change is related to the biochemical absorption characteristics of crops. It can be seen that the chlorophyll of jujube trees is damaged by the infection of leaf mites, and the first derivative spectrum has a strong sensitivity to the chlorophyll content of jujube. Consequently, hyperspectral remote sensing technology may be used to quantify the chlorophyll content of jujube under the stress of leaf mite infestation.

### SPA feature band selection

**Figure 7:**
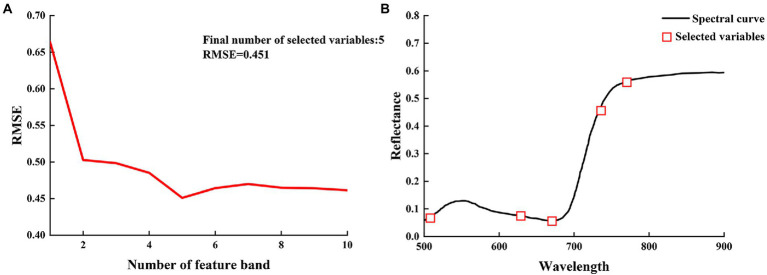
**(A)** Number of the best spectral variable for sample model; **(B)** Selection of characteristic hyperspectral bands.

Hyperspectral data are abundant in volume and wavelength information, but the correlation between wavelengths is excessively high and contains a substantial quantity of duplicated information, which poses a barrier to the storage and processing of huge amounts of data for practical applications (Liu et al., 2021). However, duplicate information in the spectral can be avoided by using a successive projection algorithm (SPA) for analysis to select the wavelengths of interest. The RMSE is calculated as the square root of the sum of the square of the departure of the observed value from the actual value divided by the number of observations and is used to assess the deviation between the observed and true values (de Sousa Fernandes et al., 2016). Given that the objective of feature wavelength extraction is to accurately categorize healthy and unhealthy plants, the fewest possible feature wavelengths should be used. In the present study, the RMSE decreased with an increase in the number of feature bands extracted (Figure 7A). The RMSE was smallest (0.451) with five feature wavelengths; the minimum RMSE value is attained when the number of bands contained in the corresponding optimal band set, which is the optimal subset of bands for the period, attains its minimum. Therefore, five characteristic wavelengths were chosen as the optimal outcome. The selected characteristic bands comprised 512.1, 628.8, 674.2, 736.6, and 773.2 nm (Figure 7B).

### Model building and prediction

An ELM is a feed-forward neural network with a single or multiple hidden layers. Unlike in conventional neural networks with back propagation (BP), the parameters of the nodes in the hidden layers of ELM are randomly assigned and never tuned. It solves the shortcomings of classic neural networks, such as sluggish training rate, local optimum instability, and sensitivity to learning rate (Li W. et al., 2020). However, the conventional ELM architecture is considered to have drawbacks (Zhang et al., 2022), such as the unpredictability of weights and thresholds, and the uncertainty of network parameters, which make it less effective at processing data and result in overfitting phenomena that reduce the accuracy of the prediction model. To optimize the parameters, such as weights and thresholds, of the ELM model in order to increase the prediction accuracy of the model, PSO was implemented (Kaloop et al., 2019). The position and velocity of the particles were updated according to Equations (6), (7), the particle fitness value was recalculated, the individual extremes and population extremes were determined with each update, and iterations were repeated in order to conduct an optimization search in the solution space.


(6) $$\begin{array}{l} V_{kd}\left(t+1\right)=\omega V_{kd}+c_{1}r_{1}\left[\mathrm{Pbes}t_{kd}\left(t\right)-X_{kd}\left(t\right)\right]\\ \;+c_{2}r_{2}\left[\mathrm{Gbes}t_{kd}\left(t\right)-X_{kd}\left(t\right)\right] \end{array}$$

(7) $$X_{kd}\left(t+1\right)=X_{kd}\left(t\right)+V_{kd}\left(t+1\right)$$

where$V_{kd}\left(t+1\right)$ is the velocity of particle k in the dth dimension in the t+1th iteration; ω is the inertia weight, generally taken to be 0.9; $c_{1}$ and $c_{2}$ are learning factors; $r_{1}$ and $r_{2}$ are random numbers in the range [0, 1]; and $\mathrm{Pbes}t_{kd}\left(t\right)$ and $\mathrm{Gbes}t_{kd}\left(t\right)$ denote the extreme positions of particle k in the individual and the population.

**Figure 8:**
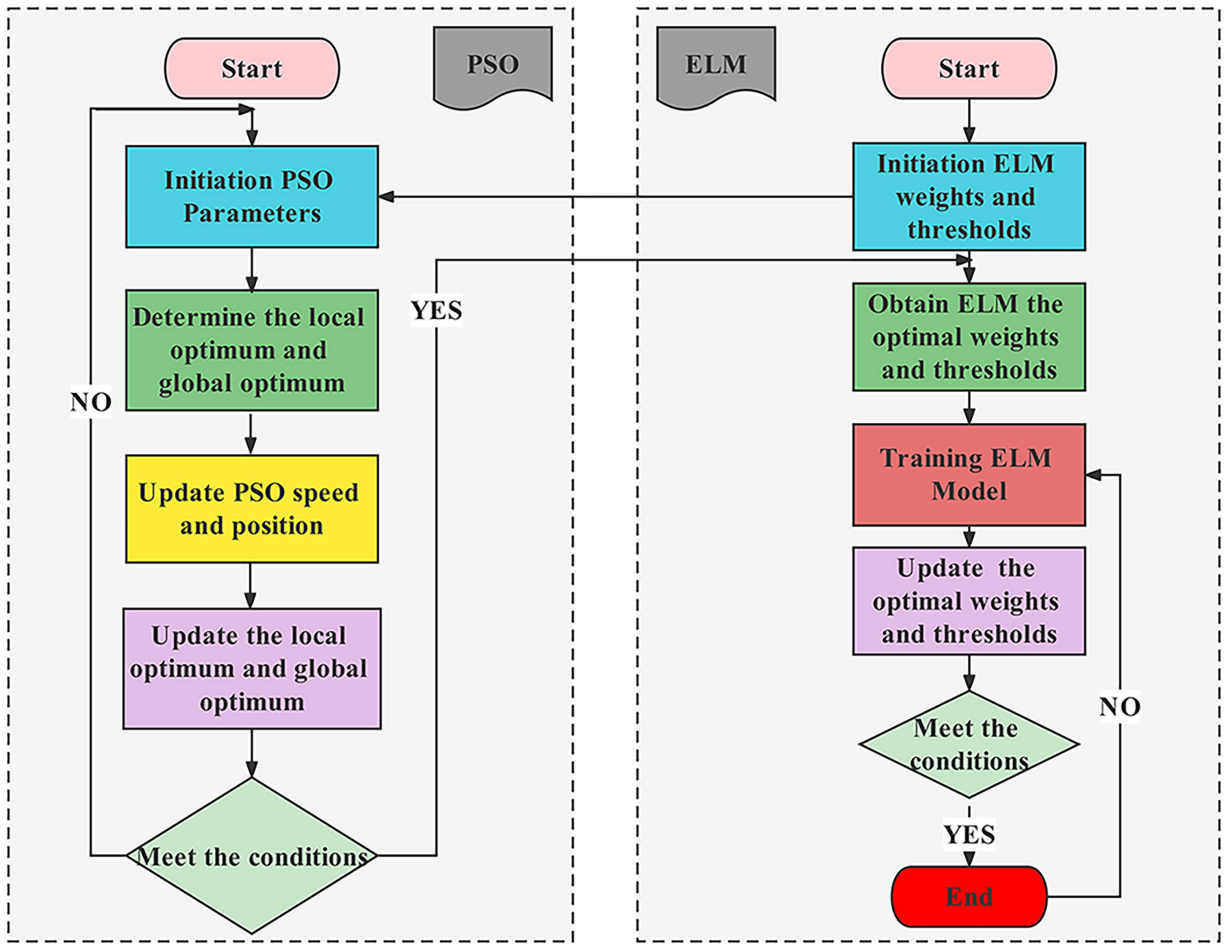
Flow chart of the PSO-ELM algorithm.

In the present study, PSO was used to improve the input weights and thresholds of the ELM model, and each particle may be considered to be an ELM model for the prediction of chlorophyll content. The location information of the particles is utilized to represent the input weights and thresholds of the ELM model (as shown in Figure 8), whereas the particle dimension *D* and the *k*th particle *k* are represented as follows:


(8) $$D=t\left(n+1\right)$$

(9) $$\theta^{k}=\left[\begin{array}{l} \omega_{11}^{k},\omega_{12}^{k},\cdots \omega_{1t}^{k},\omega_{21}^{k},\omega_{22}^{k},\cdots \omega_{2t}^{k},\cdots ,\\ \;\omega_{n1}^{k},\omega_{n2}^{k},\cdots \omega_{\mathrm{nt}}^{k},b_{1}^{k},b_{2}^{k},\cdots b_{t}^{k} \end{array}\right]$$

where n and t are the number of neurons in the input and hidden layers, respectively; $\omega_{ij}^{k}$ and $b_{j}^{k}$ are the input weights and hidden layer thresholds, respectively, and both are random numbers within the range [−1, 1], 1 < *i* < *n*, and 1 < *j* < *t*.

The PSO-ELM employs the SPA extracted characteristic bands as the independent variable and the leaf chlorophyll content of the jujube canopy as the dependent variable. Initially, the PSO parameters were initialized and the ideal fitness function value was chosen based on the performance of the PSO-ELM model. The inertia weights were set to 0.90, the maximum number of iterations was set to 100, and the learning factors were set to 1.40. Subsequently, the input weights and thresholds corresponding to each particle were substituted into the ELM model, and the predicted and measured values of RMSE were used for adaptation of the PSO to calculate the individual and global extremes. Lastly, the particle positions and velocities were updated by iterative comparison, and the particle adaptation values were calculated, and the particle extremes and global extremes were updated until the minimum error was achieved or until the maximum number of iterations was attained.

**Figure 9:**
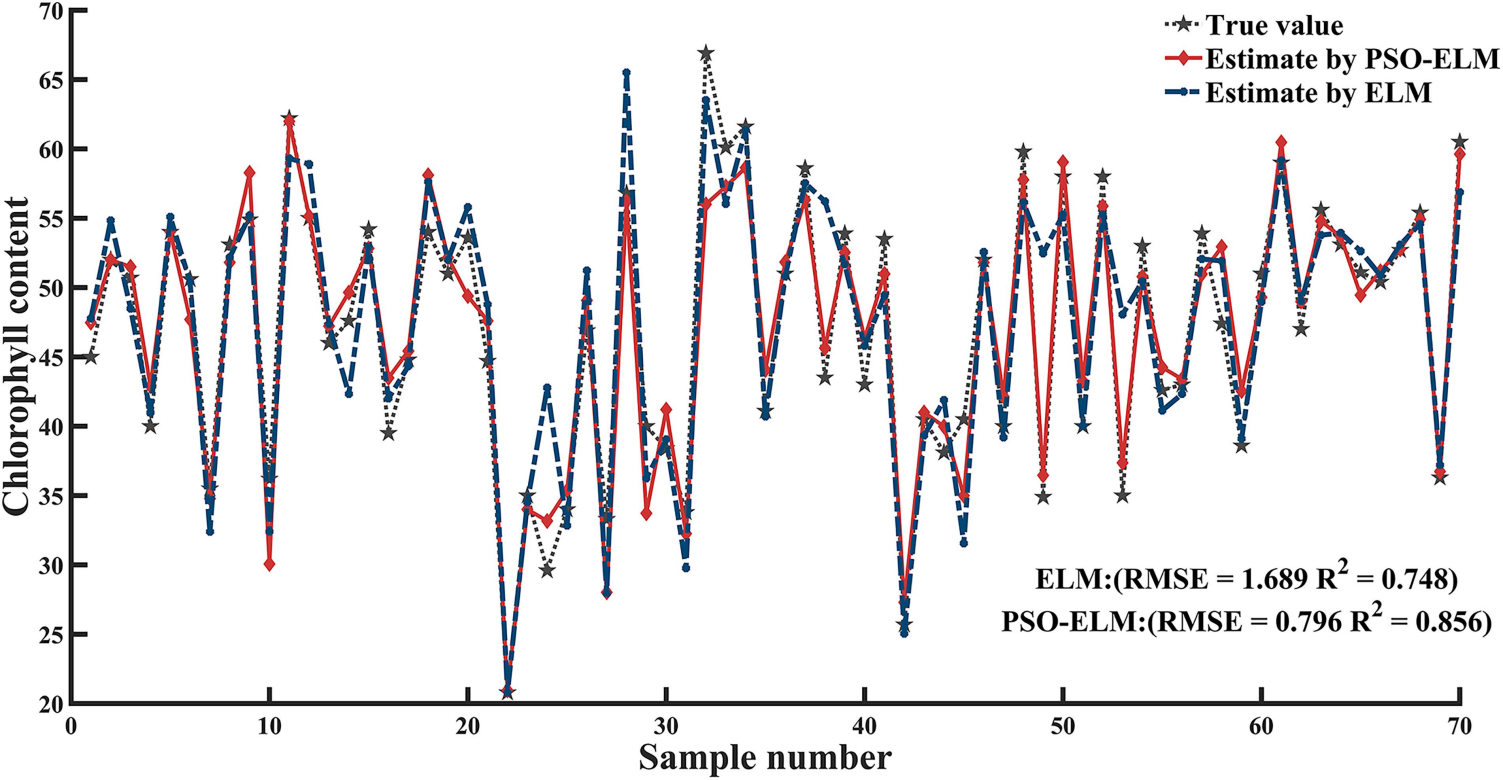
ELM and PSO-ELM models Chlorophyll content inversion model.

Using the 512.1, 628.8, 674.2, 736.6, and 773.2 nm bands as independent variables and the chlorophyll content as a dependent variable with ELM and PSO-ELM, respectively, the SPA method was utilized to create models for prediction of the chlorophyll content of jujube trees (as shown in Figure 9). The unoptimized ELM and PSO-ELM prediction values were utilized to compare and evaluate the actual measured data in order to confirm the prediction accuracy of the suggested models. Table 4 shows the prediction results of the PSO-ELM inversion model of jujube tree chlorophyll content used in this study were superior to those of the inversion model built with the simple extreme learning method, and the PSO-ELM model of chlorophyll content inversion (*R*^2^ = 0.856, RMSE = 0.796) was superior to that of the chlorophyll content inversion built with the single ELM (*R*^2^ = 0.748, RMSE = 1.689).

**Table 4:** Model comparison.

Model	Modeling set		Validation set	
	*R* ^2^	RMSE	*R* ^2^	RMSE
ELM	0.748	1.689	0.681	1.566
PSO-ELM	0.856	0.796	0.825	0.862

Given that the absolute value of the correlation between reflectance and chlorophyll content in the 500–900 nm band is generally between 0.5 and 0.65, and that there is a connection between distinct bands in this range, extracting and establishing the chlorophyll content inversion is complicated. SPA is used in this study to extract the distinctive bands of chlorophyll content inversion in order to reduce the complexity of spectral data. The number of bands is decreased to 5 after screening the contribution value, and the spectral wavenumber is lowered by 88.89%. The RMSE is 0.451. The correlation coefficients for the ELM and PSO-ELM inversion models were found to be 0.748 and 0.856, respectively. The preferential selection of five feature band parameters of SPA reduces the problem of redundancy among spectral data, improves modeling efficiency and operational efficiency, and reduces the effect of covariance of input data parameters, indicating that SPA is a more effective method for feature wavelength extraction. The sensitive bands of chlorophyll content response of jujube were selected by using SPA, and an extreme learning machine inversion model based on particle swarm optimization was established with a view to achieving rapid, accurate, and nondestructive diagnosis of canopy chlorophyll content under leaf mite infestation and improving inversion accuracy.

**Figure 10:**
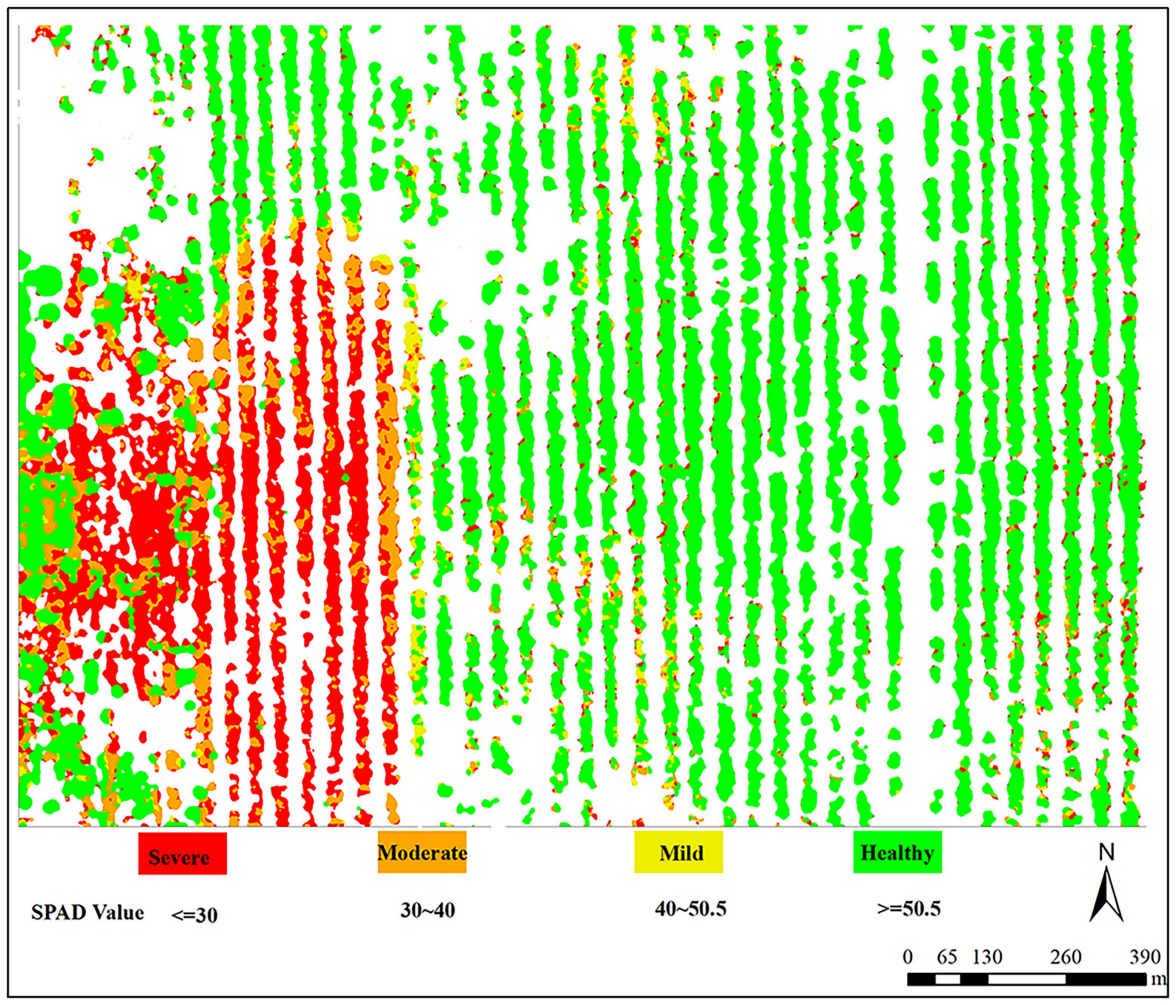
Inversion spatial distribution map of infestation severity of jujube mites.

The spatial distribution of jujube leaf mites in the research region was determined, using ArcGIS software based on the disease grading criteria for leaf mite severity (I–IV; as shown in Figure 10). The map displays the range of SPAD values that correlate to the severity of each mite infestation. While other portions of the plot were less damaged and could be mildly treated for prevention to fulfill the demands of normal jujube tree development, the left area of the plot required concentrated spraying of pesticides since it was more heavily infested. The results demonstrated that the outcomes of the ground survey and the UAV images are similar.

## Discussion

### Analysis of the correlation between spectral reflectance and chlorophyll content

Using hyperspectral spectra the benefit of “image-spectrum integration,” we acquired hyperspectral images of the jujube tree, sought to inversion of chlorophyll content under the stress of leaf mite for jujube. Recent studies have focused greater attention on the spectral properties of crop diseases, and less on the physiological and biochemical alterations imposed by the diseases. The present results revealed that leaf mite infestation influences the spectral reflectance of the jujube tree canopy, and that SPAD values are strongly associated with the leaf mite infestation index. Given the relative decrease in chlorophyll content caused by insect damage, the spectral properties of jujube plants varied significantly with severity of insect damage. As the population of leaf mites peaks, the chlorophyll content in the leaves declines, resulting in a reduction in the photosynthetic activity of the leaves and a considerable decrease in spectral reflectance. The “white patches” or yellowing of branches caused by mite feeding on the leaves decreased the leaf area index and leaf chlorophyll content of jujube. In addition, it was demonstrated that crop pests and chlorophyll are strongly associated, and that spectral data can reflect changes in chlorophyll content caused by agricultural pests. Future work will focus on transferring such an integrative methodology presented here to other agronomic parameters estimation.

### Spectral-based inversion model of chlorophyll content

In recent years, the link between reflectance spectral characteristics and pest parameters has been investigated using spectral data, and the sensitive wavebands following pest damage have been screened to enable pest monitoring and identification by classification. In the present study, we estimated the relative chlorophyll content of jujube trees under leaf mite infestation using UAV hyperspectral inversion and proposed a model for prediction of the chlorophyll content of jujube using PSO-ELM. The influence of random parameters of the ELM model on prediction accuracy and its weak generalization performance were effectively compensated. In addition, the inversion accuracy of jujube tree chlorophyll content was improved. The present results serve as a reference for the utility of UAV remote sensing for diagnosis and monitoring of leaf mite infestation in jujube.

### Challenges and prospective research

Collaborative “air–sky–ground” building of pest and disease monitoring research. In studies utilizing UAV remote sensing to monitor crop development, pests, and diseases, the determination coefficients (inversion accuracy) of the parameter inversion findings are typically greater than those of satellite remote sensing (Adao et al., 2017). However, the essential research methodologies and fundamental concepts of both are identical or comparable (Aasen et al., 2018). The essence of the higher inversion accuracy of UAV remote sensing is as follows. First, given the lower altitude of aerial photography, the distance to the crop canopy is shorter, hence there is less distortion and sensitivity of the acquired information (e.g., image texture features, spectral features, and thermal radiation features), which more accurately reflect small changes in the crop phenotypes. Second, the small spatial scale of UAV remote sensing not only objectively excludes heterogeneous factors (such as climate variation, soil conditions, moisture conditions, crop varieties, pest and disease stress, and human management practices) that affect the inversion of crop growth, pests, and diseases at medium and large scales, but also allows for the precise control of variable factors required for the experiment. However, this advantage of UAV remote sensing is also a constraint to its application (Delavarpour et al., 2021; Wang et al., 2022). Although the combination of ground-based data with UAV remote sensing data may provide point-to-point inversion of crop growth, pests, and diseases, a number of limitations remain. The geographical extent is confined to the field size, and the consequent localization and individual variability in crop phenotypes limit the portability of monitoring models based on UAV remote sensing (Rezwan and Choi, 2022), so it is impossible to duplicate the inversion laws observed at larger scales or other sites. It is challenging to overcome regional disparities in numerous elements, such as crop types, natural environmental conditions, and human management practices, using satellite remote sensing (Messina and Modica, 2020; Zhou et al., 2020). It is also challenging for satellite remote sensing to overcome the impact of the diverse inversion influences on the inversion precision. Given the restricted geographical extent, UAV remote sensing is able to effectively screen diverse information. While employing satellite remote sensing techniques, we provide UAV remote sensing data as a crucial correction index for satellite remote sensing inversion agricultural growth, pest and disease studies to aid in the development of crop models. This may provide jujube pests monitor new ideas for follow-up studies.

## Conclusion

In this study, leaf mite damage was monitored using an UAV platform equipped with a hyperspectral sensor. By acquiring hyperspectral images of jujube orchards with varying severities of leaf mite infestation, hyperspectral inversion was investigated to assess the relative chlorophyll content of jujube trees under the stress of leaf mite infestation. The results confirmed that the SPAD values of jujube plants were negatively correlated with severity of leaf mite infestation and leaf damage. Significant spectral variation was observed, with SPAD values diminished in the green peaks and red troughs of the spectral band with an increase in the severity of leaf damage. The differences in spectral reflectance among leaf mite-infested jujube plants were more pronounced. A strong correlation was observed between the SPAD value of jujube trees and the original and first-order derivative spectral reflectance of the canopy of jujube trees infested with leaf mites. It is therefore possible to quantify the leaf chlorophyll content of jujube trees under the stress of leaf mite infestation using hyperspectral remote sensing, thus providing a theoretical foundation for monitoring leaf mite infestation of jujube trees using hyperspectral remote sensing. Five feature bands were extracted using SPA: 512.1, 628.8, 674.2, 736.6, and 773.2 nm. The PSO-ELM model was developed using the extracted characteristic bands as input variables and the chlorophyll content of jujube trees as the output variable. The superior performance of the PSO-optimized ELM model demonstrated the viability of UAV deployment to perform hyperspectral inversion of the chlorophyll content of jujube plants infested with leaf mites. Thus, the variation in leaf chlorophyll content may be utilized to examine the categorization of jujube plants by severity of leaf mite infestation based on the variation in spectral characteristics.

## Data availability statement

The original contributions presented in the study are included in the article/Supplementary material, further inquiries can be directed to the corresponding author.

## Author contributions

HQ conceptualized the experiment, selected the algorithms, collected and analyzed the data, and wrote the manuscript. JM, YW, and WC trained the algorithms. HN, ZW, and PW collected and analyzed data, and wrote the manuscript. QZ, JL, and YL supervised the project. All authors contributed to the article and approved the submitted version.

## Funding

This study was supported by Laboratory of Lingnan Modern Agriculture Project (NT2021009), Basic and Applied Basic Research Project of Guangzhou Basic Research Plan in 2022 (grant no. 202201010077), The 111 Project (D18019), Intelligent agriculture integration demonstration (Y951050), and Construction of Geological Big Data of Mountain-Building Zone Along the Silk Road (E2D6050100).

## Conflict of interest

The authors declare that the research was conducted in the absence of any commercial or financial relationships that could be construed as a potential conflict of interest.

## Publisher’s note

All claims expressed in this article are solely those of the authors and do not necessarily represent those of their affiliated organizations, or those of the publisher, the editors and the reviewers. Any product that may be evaluated in this article, or claim that may be made by its manufacturer, is not guaranteed or endorsed by the publisher.
