# Supplementary material for: Inversion of chlorophyll content under the stress of leaf mite for jujube based on model PSO-ELM method
Source: Front Plant Sci. 2022 Sep 30;13:1009630. doi: 10.3389/fpls.2022.1009630 (PMC9562855; doi:10.3389/fpls.2022.1009630)
Supplement: Supplementary file 1 [file Data_Sheet_1.ZIP › Supplementary_revise_1009630/Supplementary_Material.docx]

Supplementary Material

# Supplementary Tables

**Supplementary TABLE 1 Classification of severity of jujube leaf mite infestation**

| **Classification** | **Norm** |
| --- | --- |
| **I** | Without leaf mites, leaves are spreading. |
| **II** | With a few leaf mites, the leaves are slightly creased or curled. |
| **III** | With a lot of leaf mites, silk webbing is present between the veins of the leaves. |
| **IV** | With a significant number of leaf mites, leaves have “white spots” or appear yellowish on branches. |
