# Supplementary figures and images for: Inversion of chlorophyll content under the stress of leaf mite for jujube based on model PSO-ELM method
Source: Front Plant Sci. 2022 Sep 30;13:1009630. doi: 10.3389/fpls.2022.1009630 (PMC9562855; doi:10.3389/fpls.2022.1009630)

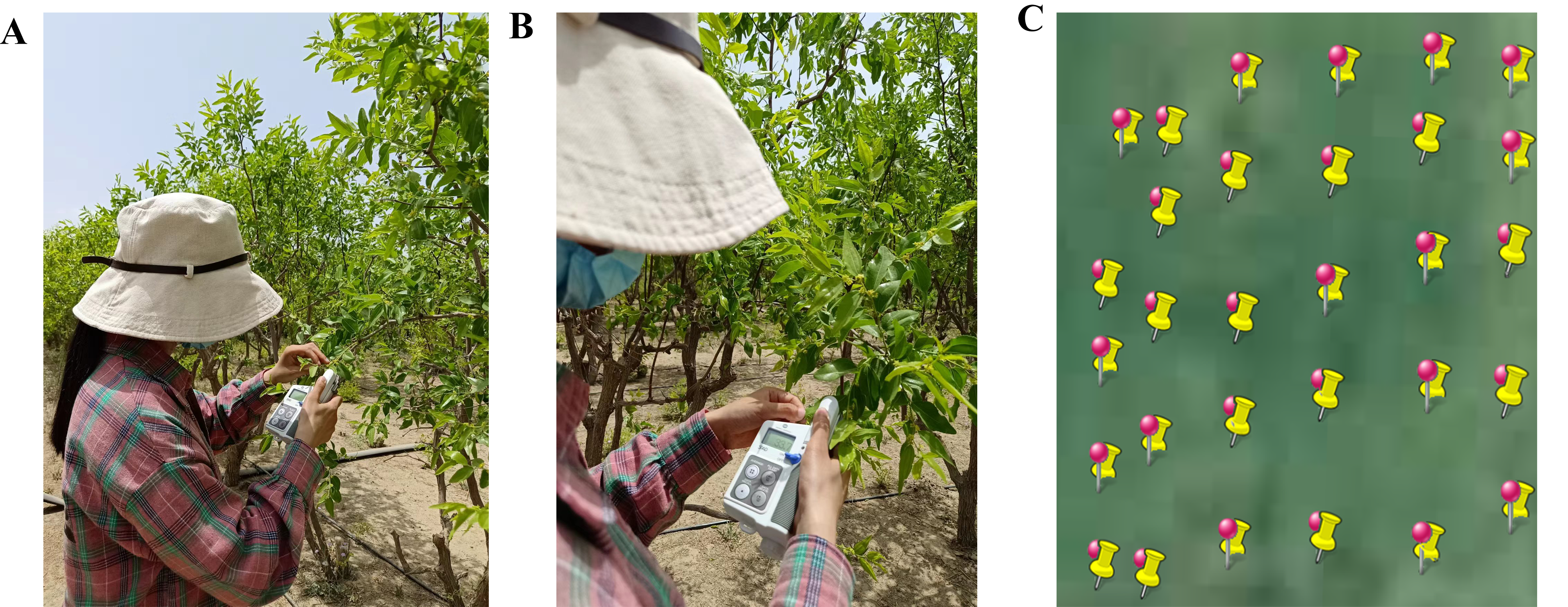

Supplement: Supplementary file 1 [file Data_Sheet_1.ZIP › Supplementary_revise_1009630/Supplementary FIGURE 2. Field collection. (A) and (B) SPAD feature parameter acquisition; (c) distribution map of one of the field layouts, three fields in total.tif]

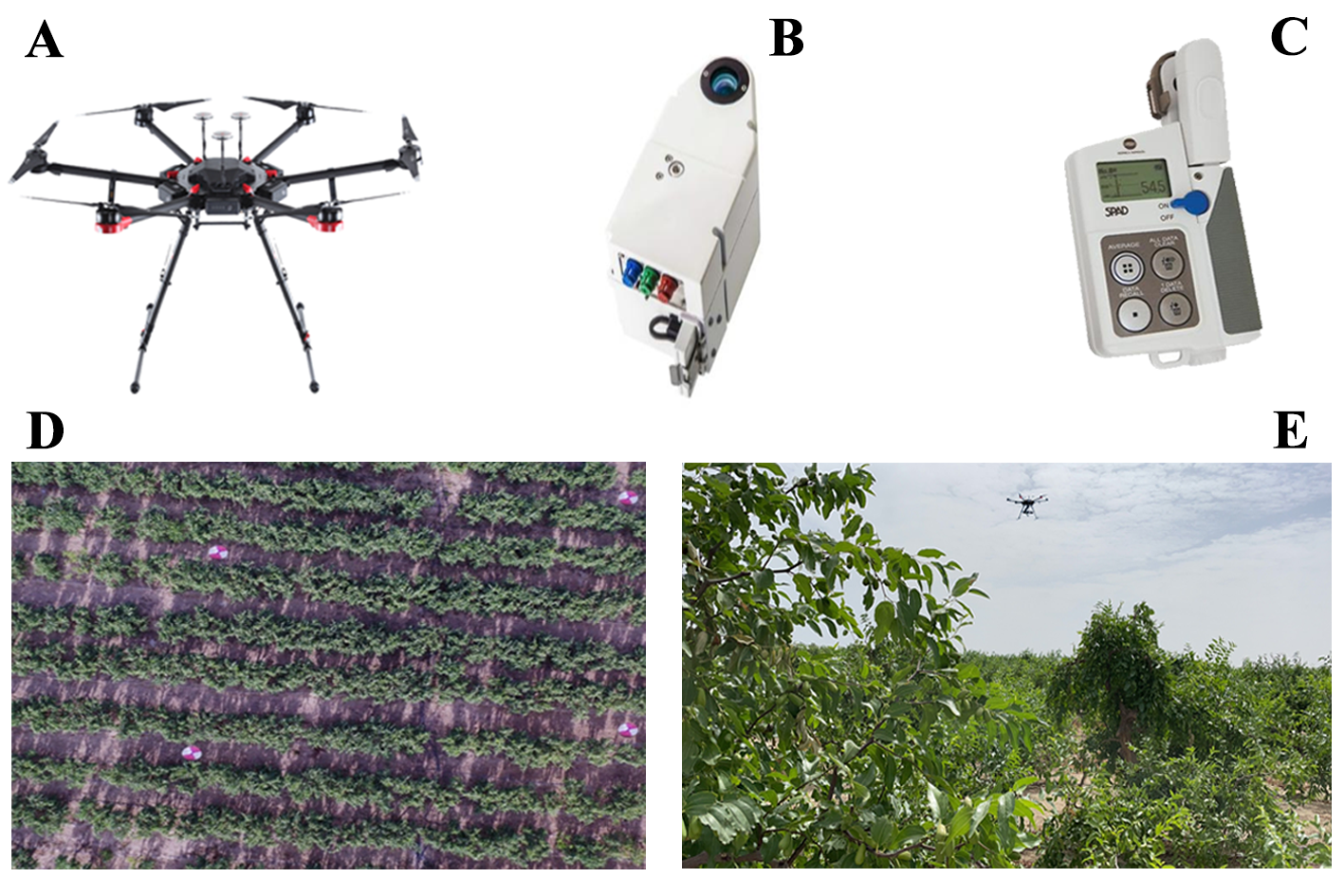

Supplement: Supplementary file 1 [file Data_Sheet_1.ZIP › Supplementary_revise_1009630/Supplementary FIGURE 1.Experimental Scenes and Device Diagrams.(A) DJI UAV M600Pro; (B) Rikola Hyperspectral Imager and(C) SPAD-502Plus;(D)and(E)Experimental Scenes.tif]
